# Menthol

**Published:** 1885-04

**Authors:** 


### Menthol.

"I think," says the Louisville *Medical News*, "it was Mr. Malcolm Morris, who, some time ago, spoke of the antiseptic use of menthol in ringworm of the scalp. I applied a solution in rectified spirit to the tinea tonsurans of a young friend in the winter of 1879, and cure resulted; and recently I have met with success in this disease from a pomade of menthol, iodoform, and vaseline. In facial neuralgia, in some form of sciatica, in neuralgic headache (clavus), and in toothache, the most recent instance being in a very severe case of a lady recovering from acute alcoholism, I have repeatedly found relief to follow within a few minutes of its application. The cones or sticks are useful for external application. Frequently, however, I use now the following formula: menthol, 30 grains; and spirit of rosemary, rectified spirit, each two drachms; or compound spirit of lavender may be used instead of the rosemary for application to the cavity of a carious tooth. I have only taken it once internally myself, but never have prescribed it. This has, however, been done."

